# Immunohistochemical and virological features of HTLV-1-associated myosites: a study of 13 patients from West Indies and Africa

**DOI:** 10.1186/1742-4690-8-S1-A41

**Published:** 2011-06-06

**Authors:** Marion Desdouits, Olivier Cassar, Thierry Maisonobe, Alexandra Desrames, Achille Aouba, Olivier Hermine, Jacqueline Mikol, Marc Polivka, Isabelle Penisson-Besnier, Pascale Marcorelles, Fabien Zagnoli, Thomas Papo, Arnaud Lacour, Zahir Amoura, Julien Haroche, Patrick Chérin, Antonio Texeira, Anne-Sophie Morin, Franck Mortreux, Eric Wattel, Michel Huerre, Marie-Christine Cumont, Huot Khun, Sylviane Bassot, Sandra Martin-Latil, Graham Taylor, Antoine Gessain, Simona Ozden, Pierre-Emmanuel Ceccaldi

**Affiliations:** 1 Epidemiology and Physiopathology of Oncogenic Viruses Unit, CNRS 3015, Pasteur Institute, Paris, France; 2 Pitié Salpêtrière Hospital, Paris, France; 3 Necker Hospital, Paris, France; 4 Lariboisière Hospital, Paris, France; 5 University Hospital Center, Angers, France; 6 University Hospital Center, Brest, France; 7 Clermont Tonnerre Army Hospital, Brest, France; 8 Bichat Hospital, Paris, France; 9 University Hospital Center, Lille, France; 10 Beaujon Hospital, Clichy, France; 11 Oncology and Biotherapies Unit, Lyon University, Lyon, France; 12 Histotechnolgy and Histopathology Unit, Pasteur Institute, Paris, France; 13 Faculty of Medicine, Imperial College, London, UK; 14 Paris 7 University, Paris, France

## Background

HTLV-1 is associated with the onset of various inflammatory diseases such as HTLV-1 Associated Myelopathy / Tropical Spastic Paraparesis, uveitis, infective dermatitis or inflammatory myopathies. Here, we aimed to get new insights into the pathogenesis of HTLV-1 associated inflammatory myopathies (HAIM) by studying muscle biopsy specimens and blood samples from 13 HAIM patients.

## Results

Mean age of patients was 52.2 years. 7 patients suffered from polymyositis (PM), and 6, from inclusion body myositis (IBM). Histopathological changes were mild to moderate in most patients. Tumor necrosis factor (TNF)-alpha and myeloid dendritic cells were detected in several patients’ biopsies, and Human Leukocyte Antigen (HLA)-ABC, HLA-DR, and matrix metalloproteinase (MMP-2, -9), in most of them. Perforin was frequently detected but there were no apoptotic myonuclei. By means of in situ hybridization, we detected rare HTLV-1 infected infiltrating cells in the muscle tissue of 4 patients. The virus belonged to the cosmopolitan A subtype, transcontinental subgroup. Plasma anti-HTLV-1 antibodies titers were high, but the proviral load was not elevated when compared to asymptomatic HTLV-1 carriers. Myositis-associated autoantibodies were found in patients with HAIM as well as in HTLV-1 infected controls without HAIM, whereas IFN-gamma plasma levels were elevated in HAIM patients.

## Conclusions

We describe 13 cases of HTLV-1 associated myositis, which show the classical anatomopathologic features of idiopathic myositis, with moderate muscle inflammation and atrophy. Proviral load was not elevated, but anti-HTLV-1 antiboides titer and IFN-gamma plasma levels were raised.

